# A comparative investigation on structure evolution of ZrN and CrN coatings against ion irradiation

**DOI:** 10.1016/j.heliyon.2019.e01370

**Published:** 2019-03-19

**Authors:** Zhengtao Wu, Yiming Wu, Qimin Wang

**Affiliations:** a School of Electromechanical Engineering, Guangdong University of Technology, 510006 Guangzhou, China; b Department of Physics (IFM), Linköping University, SE-581 83 Linköping, Sweden

**Keywords:** Materials science, Nuclear engineering

## Abstract

Binary ZrN and CrN nanostructured coatings deposited by magnetron sputtering were irradiated with 600 keV Kr^3+^ at room temperature. The ion irradiation fluences varied from 0 to 1×10^17^ Kr^3+^/cm^−2^. The results indicate the microstructure of the CrN illustrates higher stability during the Kr^3+^ ion irradiation compared to that of the ZrN. The ion irradiation produces surface etching of the CrN coating. However, the etching transfers to recrystallization and grain coarsening on the ZrN coating surface as the Kr^3+^ fluence increases.

## 1 Introduction

Nuclear reactors fuelled with (U, Pu)O_2_ are wildly used in Europe [1, 2, 3]. Consequently, plenty of plutonium and highly radioactive wastes are produced every year. In order to reduce the toxicity of the nuclear wastes, inert matrix fuels (IMFs) have been developed to optimize the burn up of the nuclear fuels [4, 5, 6]. The IMFs such as nitrides and carbides have been proposed to be suitable materials for fast neutronic systems owing to their relatively high melting temperature, low neutron absorption cross-section, high thermal conductivity, superior hardness and high corrosion resistance [7, 8, 9, 10]. The nitrides such as ZrN can form a solid solution with the fuels (for example (U, Zr)N and (Pu, Zr)N) and act as the inert matrixs to reduce the high fission density.

Unfortunately, sintering temperatures for bulk nitrides or carbides (such as ZrN, ZrC and TiC) are extremely high [11, 12, 13]. In addition, the grain sizes of the bulk ceramics range in dozens of micrometers. It is well known that nanocrystallization is one of the most important methods for strengthening the mechanical properties of materials [14, 15]. Therefore, there is a requirement to fabricate nanostructured nitrides under a relatively low temperature and investigate their irradiation behaviors against ion irradiation. In this study, both ZrN and CrN nanograined coatings deposited at 300 °C were irradiated. A comparative investigation on irradiation behaviors of the nanograined ZrN and the CrN against the ion irradiation was conducted.

## 2 Materials and methods

ZrN and CrN coatings were deposited on polished silicon (111) wafers and sapphire substrates by magnetron co-sputtering in N_2_–Ar mixture atmosphere (99.999% purity for each gas). Zr and Cr metallic targets (Ø 76 mm, purity 99.9%) were used for the sputtering. The targets were pre-sputtered for 10 min to remove the surface contaminants after reaching the base pressure of 5.0 × 10^−4^ Pa. During the ZrN and CrN depositions, the working pressure was kept at 140 mPa with the N_2_/(Ar + N_2_) flow ratio of 40%. The deposition temperature, time and bias were maintained at 300°C, 90 min and -120 V, respectively. The thicknesses of the ZrN and the CrN were controlled at ∼3 μm.

The nitride samples (deposited on sapphire substrates) were irradiated with 600 keV Kr^3+^ ions at room temperature in the Ion Beam Materials Laboratory at Los Alamos National Lab, using a 200 kV Danfysik High Current Research Ion Implanter. The 600 keV Kr^3+^ ions were implanted at normal incidence with an average flux of ∼1.0×10^12^ Kr^3+^/cm^2^/s. The total irradiation fluences were set at 0, 5.0×10^15^, 5.0×10^16^ and 1.0×10^17^ Kr^3+^/cm^2^, respectively.

Both normal and grazing incidence X-ray diffraction (GIXRD, Rigaku Ultima IV, Japan) tests were conducted to obtain the phase structure of the samples. An incidence angle of 3° was used during the GIXRD tests. The microstructure of the pristine ZrN and CrN was investigated by TEM observation (FEI, Tecnai G^2^ F20, U.S.). Scanning electron microscope (SEM, FEI, Nano 430, U.S.) was applied to obtain both surface and cross-sectional morphologies of the coatings. Both hardness and elastic modulus values of the coating were determined by nanoindentation tester (Anton-Paar TriTec, TTX-NHT^2^, Austria). The maximum penetration depth was set at ∼10% of the coating thickness (i.e. ∼300 nm) to minimize the influence of the indentation size effect. Both loading and unloading time of the indenter was fixed at 30 s. In order to release material creep, a 30 s pause time was applied after the loading.

## 3 Results & discussion

**Fig. 1:**
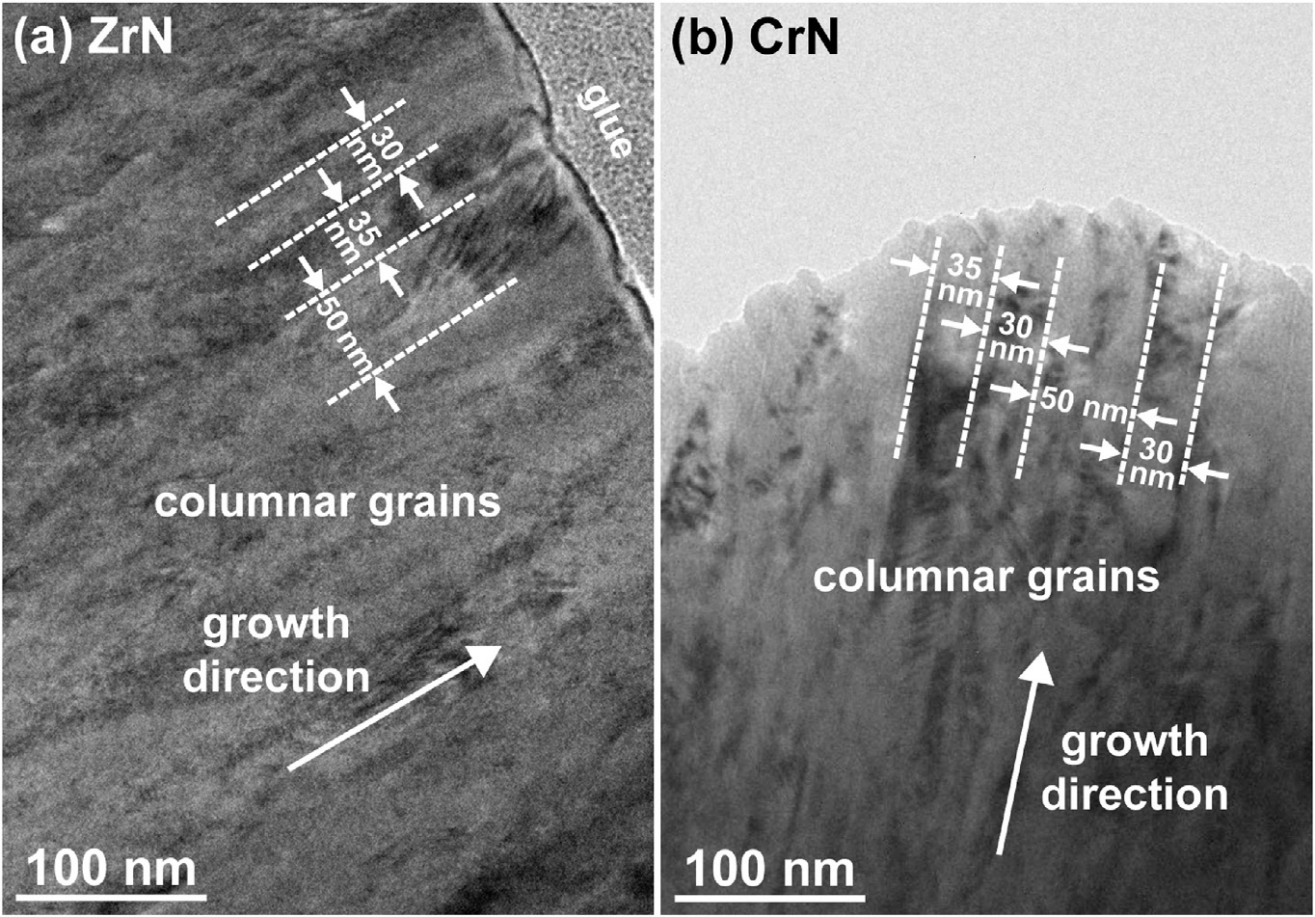
Bright field cross-sectional TEM images of the (a) ZrN and (b) CrN coatings. The solid arrows indicate the growth direction of the coating.

The TEM observation was carried out to acquire the microstructure of the coatings. Fig. 1 shows the bright field TEM images of the as-deposited coatings. An identical dense and continuous column structure can be noticed in both of the ZrN and the CrN coatings. No pores and microcracks can be observed in the coatings. In addition, the grain sizes of the ZrN and the CrN columnar grains varied in the same range of 30–50 nm. Thus, both nanostructured ZrN and CrN nitrides were fabricated.

**Fig. 2:**
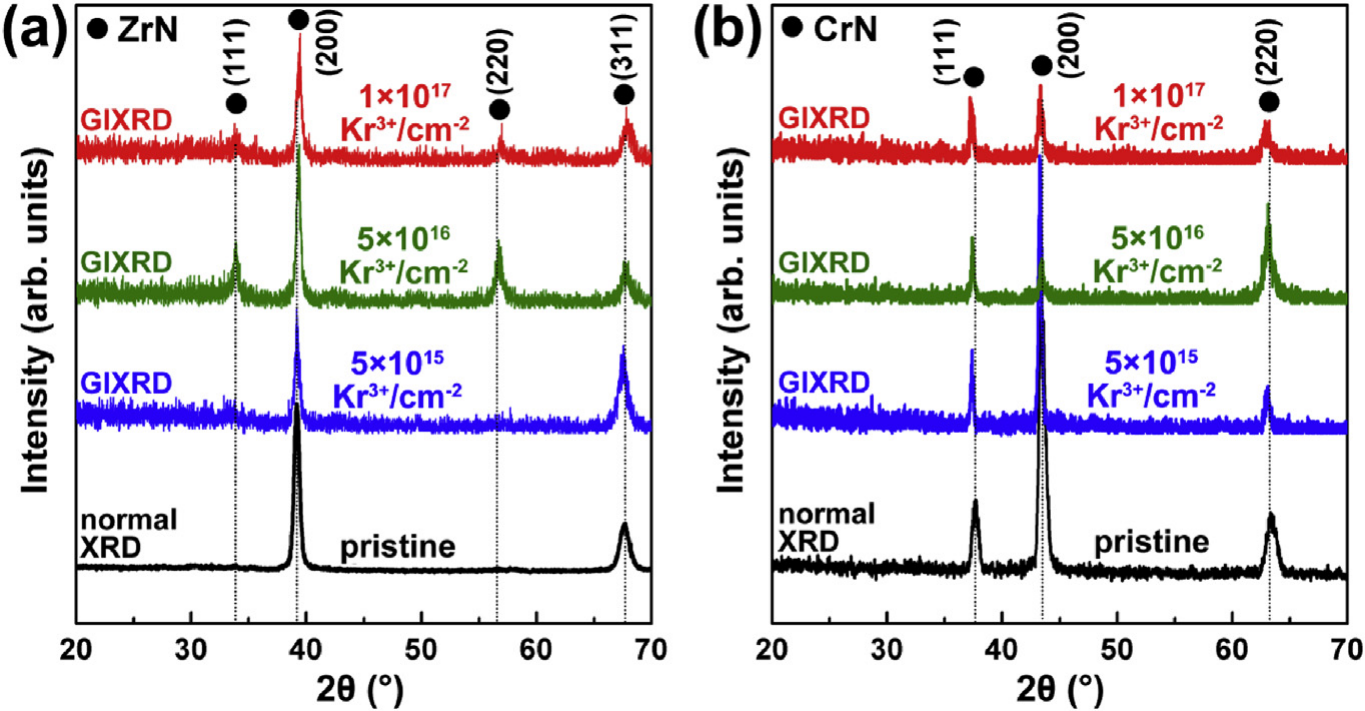
The XRD patterns of the (a) ZrN and (b) CrN coatings after the ion irradiation. The black curves indicate the normal diffraction patterns of the as-deposited coatings. The blue, green and red curves correspond to the GIXRD patterns of the coatings after the 5×10^15^, 5×10^16^ and 1×10^17^ Kr^3+^/cm^−2^ irradiation, respectively.

Fig. 2 shows the GIXRD patterns of the ZrN and CrN coatings after the 600 keV Kr^3+^ ion irradiation with varying fluence. The normal XRD was used to indicate both phase structure and preferred orientation of the pristine coatings. The GIXRD was employed to determine the phase composition of the irradiated layers. In Fig. 2(a), the pristine ZrN (JCPDS No. 35-0753) coating illustrates the typical fcc crystalline structure with the preferred orientation of (200). No reaction or phase transformation can be observed after the ZrN coating was irradiated with the 600 keV Kr^3+^. However, the right shift of the characteristic peaks of the ZrN can be found as the Kr^3+^ fluence increases. This indicates the release of internal stress occurred during the ion irradiation. For the pristine CrN (JCPDS No. 76-2494), it presents the typical fcc crystalline structure with the preferred orientation of (200). No reaction or phase transformation can be observed after the ion irradiation as well. The phase structure of the CrN illustrates high stability during the Kr^3+^ ion irradiation. However, a slightly left shifts of the characteristic peaks of the CrN can be found. The ion irradiation produced bombardment and subsequent densification (revealed by the cross-sectional observation, discussed below) of the CrN surface layer, resulting in the increasing of internal compressive stress.

**Fig. 3:**
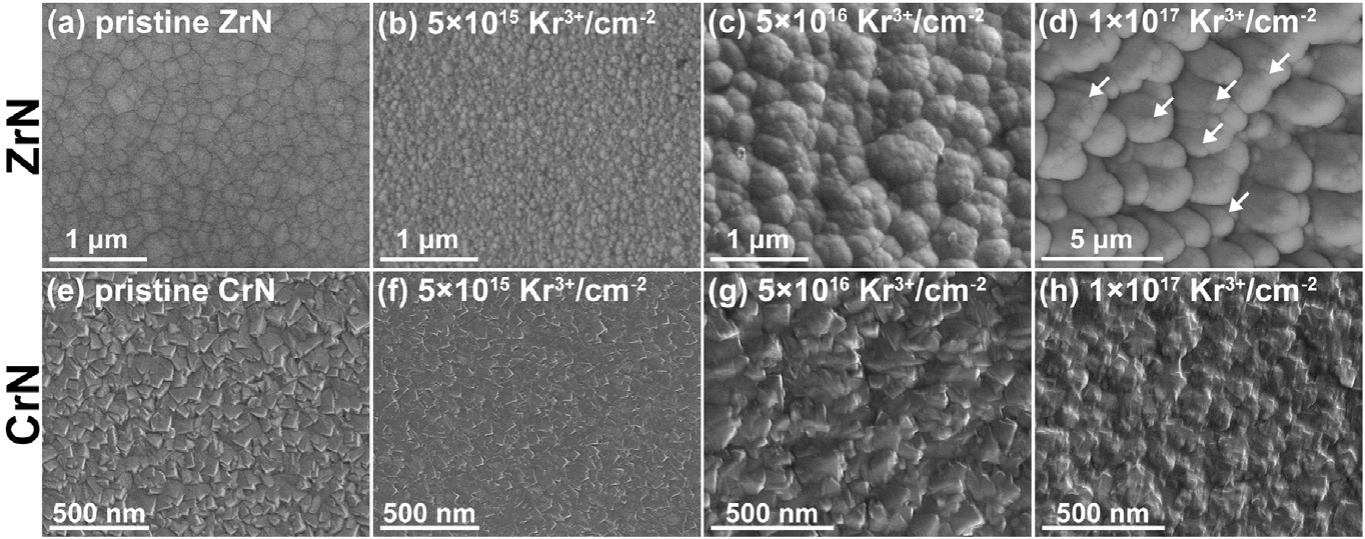
The surface SEM images of the ZrN and CrN coatings with respect to the fluence of the Kr^3+^ ion irradiation. (a) pristine ZrN, (b) 5×10^15^ Kr^3+^/cm^−2^ on ZrN, (c) 5×10^16^ Kr^3+^/cm^−2^ on ZrN, (d) 1×10^17^ Kr^3+^/cm^−2^ on ZrN, (e) pristine CrN, (f) 5×10^15^ Kr^3+^/cm^−2^ on CrN, (g) 5×10^16^ Kr^3+^/cm^−2^ on CrN, (h) 1×10^17^ Kr^3+^/cm^−2^ on CrN.

Fig. 3 exhibits the surface SEM images of the ZrN and CrN coatings after the 600 keV Kr^3+^ ion irradiation. For the ZrN, the pristine coating indicates a granular surface microstructure. After the ion irradiation with the fluence of 5.0×10^16^ Kr^3+^/cm^2^, the surface etching of the ZrN can be noticed. However, the etching transfers to recrystallization and grain coarsening on the ZrN coating surface as the ion fluence increases up to 5×10^16^ Kr^3+^/cm^2^. A larger grain size of the ZrN can be achieved with further increasing the ion fluence to 1×10^17^ Kr^3+^/cm^2^. In addition, microcracks (marked by arrows) can be observed on the surface grains of the ZrN. For the CrN, the pristine coating indicates a pyramid microstructure. After the Kr^3+^ ion irradiation with the fluence of 5.0×10^16^/cm^2^, the surface etching of the CrN can be noticed as well. Additionally, the roughness of the coating surface decreases. With further increasing the ion fluence up to 1×10^17^ Kr^3+^/cm^2^, only surface etching can be observed. This is substantially different from the irradiation behavior of the ZrN coating, which indicates the transformation from etching to recrystallization and grain coarsening. Nanoindentation tests were applied to illustrate the evolution in hardness and elastic modulus the coatings, the obtained values were shown in Table 1. Both the hardness and the elastic modulus the ZrN coating decrease with the applying of the Kr^3+^ ion irradiation, owing to the increase of the grain sizes. However, both hardness and elastic modulus of the CrN coatings change slightly after the Kr^3+^ ion irradiation. These above results reveal that the microstructure of the nanostructured CrN illustrates a higher stability during the Kr^3+^ ion irradiation comparing to that of the nanograined ZrN.

**Table 1:** Hardness (*H*) and elastic modulus (*E*) of the coatings after the 1×10^17^ Kr^3+^/cm^−2^ ion irradiation.

Samples	ZrN		CrN	
	*H*/GPa	*E*/GPa	*H*/GPa	*E*/GPa
Pristine coating	23.9 ± 1.0	380.1 ± 20.1	18.6 ± 0.6	353.2 ± 12.1
Irradiated coating	14.9 ± 4.2	215.3 ± 40.4	19.1 ± 1.8	365.8 ± 27.4

**Fig. 4:**
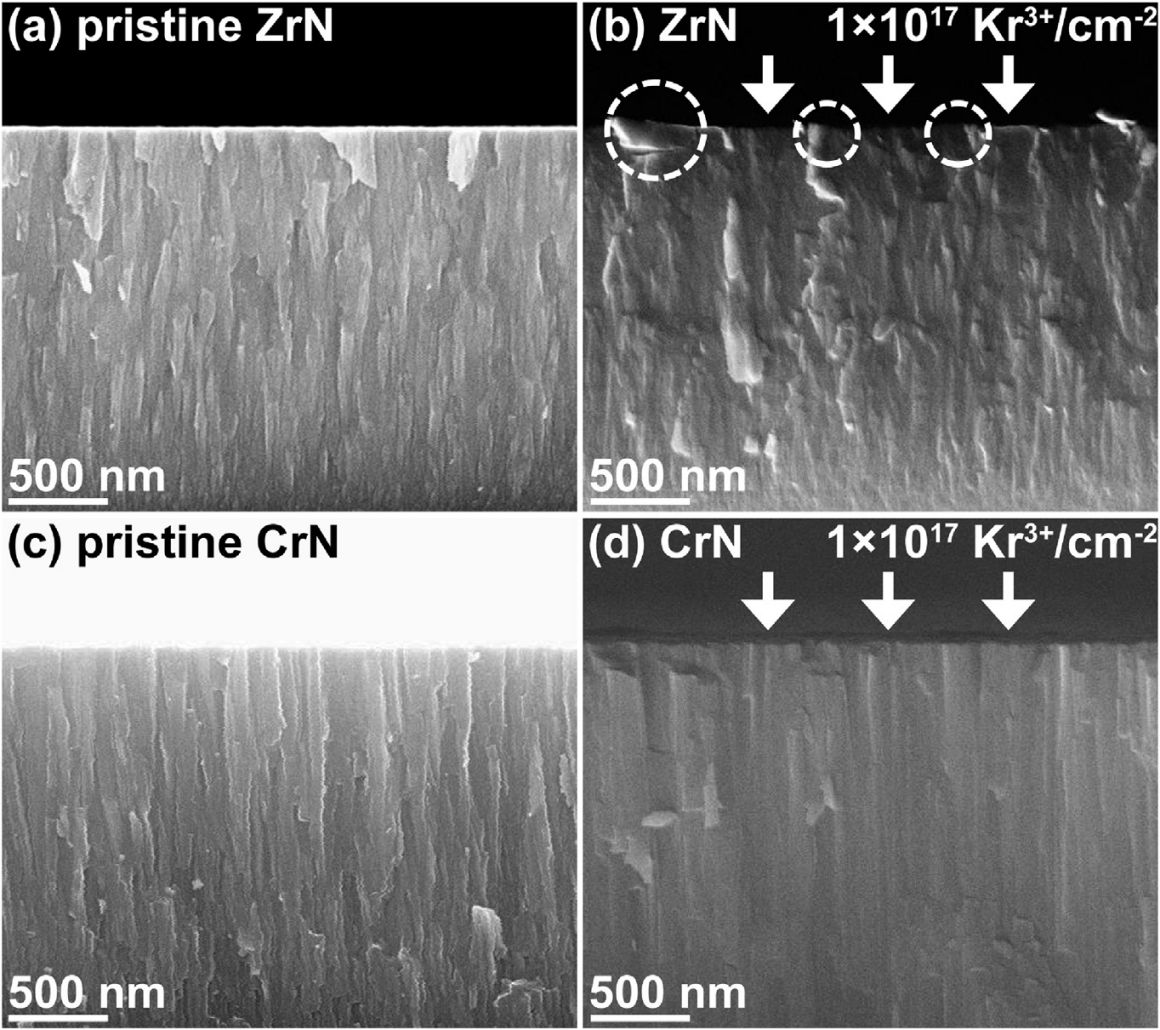
The cross-sectional SEM images of the ZrN and CrN coatings. (a) and (c) show the cross-sections of the ZrN and CrN, respectively. (b) and (d) illustrate the cross-sections of 600 keV Kr^3+^ ion irradiated ZrN and CrN, respectively. The fluence of the ions was 1×10^17^ Kr^3+^/cm^2^.

Fig. 4 shows the cross-sectional SEM images of the ZrN and the CrN coatings after the 600 keV Kr^3+^ ion irradiation with a fluence of 1×10^17^ Kr^3+^/cm^2^. No bubbles or cracks can be observed in the cross-sections of these coatings. This indicates the higher irradiation tolerance of the nanostructured nitrides compared to the bulk ceramics of materials [9, 16, 17]. The column-grains can be observed in the ZrN bottom layer. No continuous and columnar grains can be noticed in the ZrN irradiated layers. It demonstrates that the continuous column-grains were interrupted on the ZrN coating surface. In addition, protrusion (marked by circles) on the coating surface can be noticed as well. Actually, when the recrystallization and the grain coarsening of the ZrN occurred, the ZrN columnar grains will be broken. However, the cross-section of the irradiated CrN still indicates a continuous and columnar structure. The average widths of the CrN columnar grains change slightly after the ion irradiation. In addition, in Fig. 4(d), the cross-section of the irradiated CrN was much smooth compared to the pristine CrN. It reveals that the ion irradiation produced bombardment and subsequent densification of the CrN irradiated layer, resulting in the increasing of internal compressive stress (Fig. 2(b)). These above results further prove that the microstructure of the CrN illustrates a higher stability during the Kr^3+^ ion irradiation comparing to that of the ZrN. An analytical TEM investigation into the irradiated layers of the ZrN and the CrN coatings has to be conducted in future work.

## 4 Conclusions

In summary, both nanograined ZrN and CrN were irradiated with 600 keV Kr^3+^ at room temperature. The CrN shows higher structure stability against the irradiation compared to that of the ZrN. The 600 keV Kr^3+^ ion irradiation produces surface etching of the CrN coating. The ion irradiation with the fluence of 5.0×10^16^ Kr^3+^/cm^2^ produces surface etching on the ZrN coating as well. However, recrystallization and grain coarsening of the ZrN occur under the Kr^3+^ ion irradiation with a high fluence. The continuous cross-section microstructure of the ZrN was interrupted by the ion irradiation with a large fluence.

## Declarations

### Author contribution statement

Zhengtao Wu: Performed the experiments; Analyzed and interpreted the data; Contributed reagents, materials, analysis tools or data; Wrote the paper.

Yimin Wu: Performed the experiments; Analyzed and interpreted the data.

Qiming Wang: Conceived and designed the experiments; Performed the experiments; Analyzed and interpreted the data; Contributed reagents, materials, analysis tools or data.

### Funding statement

This work was supported by the National Natural Science Foundation of China (51875109), the China Postdoctoral Science Foundation (2016M600641) and the Natural Science Foundation of Guangdong Province (2018A030310546).

### Competing interest statement

The authors declare no conflict of interest.

### Additional information

No additional information is available for this paper.

## References

[1] Thetford R., Mignanelli M. (2003). The chemistry and physics of modelling nitride fuels for transmutation. J. Nucl. Mater..

[2] Líška P., Cognet G., France C. (May, 2011). The Allegro project–European project of fast breeder reactor. Proceedings of the 1st International Nuclear Energy Congress.

[3] Vaudez S., Belin R.C., Aufore L., Sornay P., Grandjean S. (2013). A new fabrication route for SFR fuel using (U, Pu) O2 powder obtained by oxalic co-conversion. J. Nucl. Mater..

[4] Shimamura K., Arima T., Idemitsu K., Inagaki Y. (2007). Thermophysical properties of rare-earth-stabilized zirconia and zirconate pyrochlores as surrogates for actinide-doped zirconia. Int. J. Thermophys..

[5] Salvatores M., Palmiotti G. (2011). Radioactive waste partitioning and transmutation within advanced fuel cycles: achievements and challenges. Prog. Part. Nucl. Phys..

[6] Terrani K.A., Snead L.L., Gehin J.C. (2012). Microencapsulated fuel technology for commercial light water and advanced reactor application. J. Nucl. Mater..

[7] Burghartz M., Ledergerber G., Hein H., Van der Laan R., Konings R. (2001). Some aspects of the use of ZrN as an inert matrix for actinide fuels. J. Nucl. Mater..

[8] Streit M., Ingold F. (2005). Nitrides as a nuclear fuel option. J. Eur. Ceram. Soc..

[9] Egeland G., Valdez J., Maloy S., McClellan K., Sickafus K., Bond G. (2013). Heavy-ion irradiation defect accumulation in ZrN characterized by TEM, GIXRD, nanoindentation, and helium desorption. J. Nucl. Mater..

[10] van Vuuren A.J., Skuratov V., Uglov V., Neethling J., Zlotski S. (2013). Radiation tolerance of nanostructured ZrN coatings against swift heavy ion irradiation. J. Nucl. Mater..

[11] Ryu H.J., Lee Y.W., Cha S.I., Hong S.H. (2006). Sintering behaviour and microstructures of carbides and nitrides for the inert matrix fuel by spark plasma sintering. J. Nucl. Mater..

[12] Xue J.X., Zhang G.J., Xu F.F., Zhang H.B., Wang X.G., Peng S.M., Long X.G. (2013). Lattice expansion and microstructure evaluation of Ar ion-irradiated titanium nitride. Nucl. Instrum. Methods Phys. Res., Sect. B.

[13] Frost B.R. (2013). Nuclear Fuel Elements: Design, Fabrication and Performance.

[14] Gleiter H. (2000). Nanostructured materials: basic concepts and microstructure. Acta Mater..

[15] Nastasi M., Parkin D.M., Gleiter H. (2012). Mechanical Properties and Deformation Behavior of Materials Having Ultra-fine Microstructures.

[16] Van Vuuren A.J., Neethling J., Skuratov V., Uglov V., Petrovich S. (2014). The effect of He and swift heavy ions on nanocrystalline zirconium nitride. Nucl. Instrum. Methods Phys. Res., Sect. B.

[17] Yang Y., Dickerson C.A., Allen T.R. (2009). Radiation stability of ZrN under 2.6 MeV proton irradiation. J. Nucl. Mater..

